# Asocial balance—how your friends determine your enemies: understanding the co-evolution of friendship and enmity interactions in a virtual world

**DOI:** 10.1007/s42001-017-0010-9

**Published:** 2017-12-22

**Authors:** Maximilian Sadilek, Peter Klimek, Stefan Thurner

**Affiliations:** 1 Section for Science of Complex Systems, Medical University of Vienna, Spitalgasse 23, 1090 Vienna, Austria; 2 Complexity Science Hub Vienna, Josefstädter Strasse 39, 1080 Vienna, Austria; 3 Santa Fe Institute, 1399 Hyde Park Road, Santa Fe, NM 87501 USA; 4 IIASA, Schlossplatz 1, 2361 Laxenburg, Austria

**Keywords:** Social network formation, Triadic closure, Social balance, Co-evolution, Multi-layer network

## Abstract

Social interactions take place simultaneously through different interaction types, such as communication, friendship, trade, exchange, enmity, revenge, etc. These interactions can be conveniently described with time-dependent multi-layer networks. Little is known about the dynamics of social network formation on single layers. How the dynamics on one layer is coupled to and influences the dynamics on another layer is a completely unexplored territory. This is mainly due to the lack of comprehensive microscopic interaction data on time-dependent multi-layer networks. In this work, we study a unique dataset of 350,000 odd players in a massive multi-player online game, for which we know practically every social interaction event. We focus on the dynamics of friendship interactions and how they are coupled to the dynamics of enmity interactions. We are able to identify the driving processes behind the joint network formation of friendship and enmity links. The essential mechanisms turn out to be specific triadic closure rules. We propose a simple dynamical model that allows us to predict not only the correct levels of social balance but also the detailed simultaneous structural properties of the friendship and enmity networks, including their degree distributions, clustering coefficients and nearest neighbor degrees. While the formation of new friendship links can be largely understood on the basis of structural features of the friendship network alone, this is not true for enmity networks. The formation of enmity links is driven by the need to socially balance triadic relations that contain negative and positive interactions. Networks of enmity relations can only be understood structurally in the context of the positive social ties they are embedded in.

## Introduction

Over the past decades, the focus of social science has shifted from topics centered around social behavior of individuals and groups to relationships and interactions among social entities. Network science and methodology from complex adaptive systems [1, 2] have become increasingly relevant for quantitative social science [3]. A central challenge of contemporary social science is to understand the structure and dynamics of social networks on the basis of “microscopic” interactions between individuals. In recent years, two developments greatly facilitated the empirical side of this task: first, electronic fingerprints and automated methods of data acquisition gradually superseded conventional methods such as interviews and questionnaires. This opened completely new scales of analysis, while eliminating various sources of bias [4]. Second, the general availability of social network data has substantially increased, not least because an increasing number of people participate in virtual worlds, such as massive multiplayer online games [5, 6].

Today, structural properties of numerous real-world social networks are well-studied. The most important structural information is carried in the distribution functions of the degrees, the clustering coefficients, and the nearest neighbor degrees. They allow us to make statements about robustness, efficiency, hierarchy and assortativity of the underlying networks. Countless findings have been reported, such as the appearance of power laws in scientific collaboration networks [7], mobile communication networks [8], or networks of co-starring movie actors [9], just to name a few. In contrast, the study of social network dynamics has been primarily approached from a purely theoretical perspective: a significant amount of literature concentrates on hypothetical dynamical models [10] and agent-based models [11]. This has led to the paradoxical situation that, on the one hand, the dynamical origin of a majority of the observed network structures is virtually unknown and cannot be related to actually observed interaction patterns. On the other hand, many proposed mechanisms of network formation lack empirical verification. To overcome this discrepancy, it is necessary to get a better understanding of the intrinsic connection between structure and actual microscopic dynamics of social networks. In other words, the following two essential questions need to be addressed: What are the actual microscopic key processes behind social network formation? And, how do these processes lead to the observed structural properties, such as degree distributions or clustering coefficients as a function of degree? This requires a thorough empirical analysis of network formation processes, as well as the testing in a model environment that incorporates these processes and is able to explain the observed network properties. Many real-world social networks are comprised of positively and negatively connoted relations and show evidence of social balance [12]. Realistic models of social network formation should be able to predict the correct levels of social balance.

In this work, we provide some answers to the above questions, based on the analysis of friendship and enmity networks obtained from the online game PARDUS [13–17]. PARDUS is a browser-based, open-ended massive multiplayer online game (MMOG) with presently more than 350,000 registered players. For a detailed discussion of the structural and dynamical properties of the game see [14]. The players live in a virtual, futuristic universe, where they act in a self-determined manner to pursue their own goals and interact with each other in a multitude of ways. Typically, players strive for increasing social status and wealth, which can be achieved by engaging in various political and economic activities. In this context, social factors such as cooperation and conflict become important. In particular, players in PARDUS can mark each other as friends or enemies. These markings persist until they are removed. Each marking is private and only known to the two players involved in the associated friendship or enmity relation. At any time, each player maintains two personal lists: one containing all their friends and enemies, and one containing all players that have marked them as a friend or enemy, respectively.

We have a full record of all friendship and enmity markings for 1235 consecutive days. From this data, we extract a time series of networks in the following way: if at any time during day *t* player *j* is marked by player *i* as a friend or an enemy, a friendship respectively enmity link from *i* to *j* is added to the network associated with day *t*. This is done for all players and days. We obtain a dataset comprising friendship and enmity networks of 4000–5000 continuously active players for 1235 days. Figure 1 schematically illustrates the two-layer network structure of friendship (green arrows) and enmity (red arrows) markings among players. Each player is represented by two nodes (grey circles connected by a dashed line).

The paper is structured as follows: first, we analyze the dynamics of network formation for the PARDUS friendship and enmity interactions, which leads to the identification of the most relevant driving processes behind the dynamics. We then implement these processes in a two-layer network model and perform numerical simulations. Finally, we validate the model by comparing its emergent network properties and social balance levels with those found in the primary data. The proposed model is not only compatible with social balance theory, but also in good agreement with the structural network properties that are found in the data.

## Identification of the dynamical driving processes

**Fig. 1:**
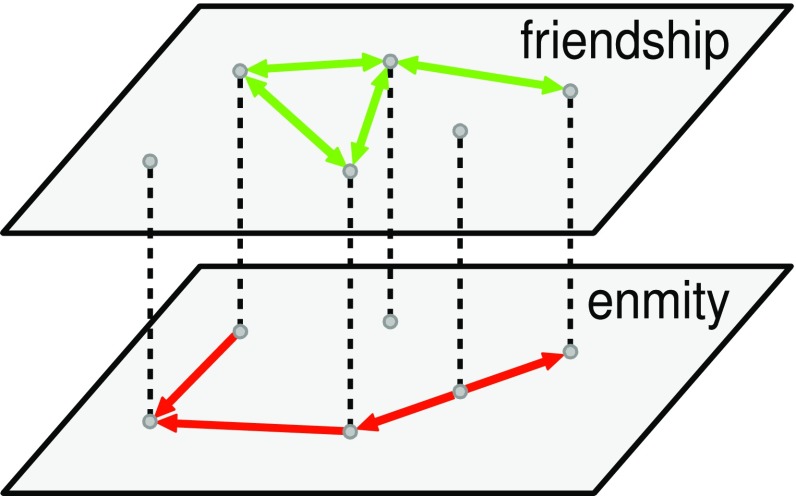
Snapshot of a two-layer social network of friendship (green arrows) and enmity (red arrows) relations. Each pair of nodes (grey circles) connected by a dashed line represents a single individual

### Basic network dynamics

**Fig. 2:**
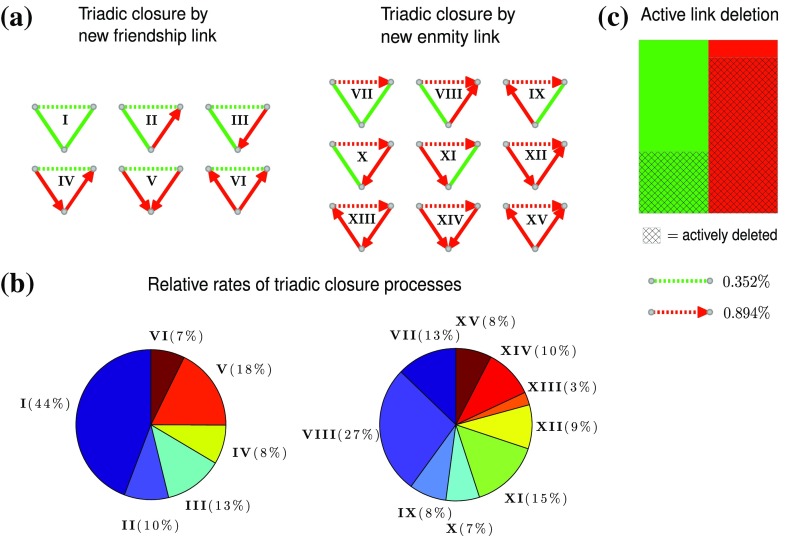
Driving processes behind two-layer social network formation, based on the numerical analysis of coupled friendship and enmity networks obtained from the massive multiplayer online game PARDUS. **a** Schematic illustration of all possible types of triadic closure processes, given that friendship links are reciprocal and enmity links are uni-directional. Shown on the left (right) are triads that are closed by a friendship (enmity) link, the respective positions of the closing links are indicated by the dashed lines. **b** Relative frequencies of triadic closure processes when new friendship (left) respectively enmity (right) links are created. Note that the rates do not add up to one because several triads can be closed simultaneously. **c** Fractions of links that are actively deleted by players. While friendship links (green) primarily disappear from the network because a player leaves the game, enmity links (red) are often actively withdrawn

Players enter and exit the game. When a player enters the game initially, there are no social interaction links. When the player eventually meets and interacts with other avatars, every interaction between them is recorded as a link in a temporal multi-layer network. We only focus on the two layers of friendship and enmity. Links in the two network layers exist until they are actively removed from the network by the players that established them, or when a player exits the game. In the latter case, the corresponding node is removed from the two-layer friendship–enmity network, and their links in both layers are deleted.

To analyze the dynamics of network formation, we look at link creation and deletion events and at player entry and exit events. Since players and their actions both have characteristic life times, the number of nodes and links in the networks can be expected to be relatively stationary. To confirm that this is indeed the case, and to later determine the link and node creation (deletion) rates, we check if and when the stationarity conditions for node and link dynamics are met. If $\Delta N^{+}$ and $\Delta N^{-}$ denote the average numbers of players entering or leaving the game within a time period of interest, and $\Delta L^{+}$ ($\Delta L^{-}$) denotes the average number of link creation (deletion) events, stationarity requires:

1 $$\begin{aligned}&\Delta N^{+}\approx \Delta N^{-}\gg \Delta N^{+}-\Delta N^{-},\end{aligned}$$

2 $$\begin{aligned}&\Delta L^{+}\approx \Delta L^{-}\gg \Delta L^{+}-\Delta L^{-} . \end{aligned}$$

We observe that after an initial growth phase, where $\Delta N^{+}>\Delta N^{-}$, both the network size *N* and the total number of links *L* fluctuate around constant values, indicating indeed a stationary state. We choose our period of observation as the final 100 days of the dataset. From Eqs. (1) and (2) the node and link creation rates can be computed, see below.

In the PARDUS society, we find that the overlap between friendship and enmity networks is negligible (Jaccard coefficient $J\sim 0$), and their respective reciprocities are fundamentally different ($\rho > 0.55$ for friendship and $\rho < 0.13$ for enmity). Therefore, the typical interaction between two players is either mutual and friendly or asymmetric and hostile [13, 14].

### Triadic closure dynamics

We now show that the creation of new links can be mainly attributed to two types of triadic closure processes, and that players actively delete their enmity links much more frequently than their friendship links. According to social balance theory, the link creation dynamics is determined by the local network structure [18, 19]. In particular, links tend to be created between nodes that share a common neighbor, i.e., the generic process of link creation is the closure of an open triad [20, 21]. Given 2 possible link types and 2 possible link directions for each of the 3 edges, up to a factor of 2 due to mirror symmetry, there are $(2\times 2)^3/2=32$ unique ways to close an open triad. We make use of the fact that most of the friendship links are reciprocal and disregard their directionality. This reduces the number of possibilities. Along a similar line of reasoning, due to their rare occurrence, we ignore reciprocal enmity links. This leaves us with six different types of triadic closure processes if a new friendship link is created, and nine types if a new enmity link is created, see Fig. 2a.

Figure 2b shows the corresponding relative rates for our data, i.e., how often a newly appearing link closes an open triad of a specific type divided by the total number of closure events within our observation period. We find that both friendship (left) and enmity (right) link creation events are dominated by one type of triadic closure process. Type *I* is responsible for ($44\%$ of the created friendship links, type *VIII* for $27\%$ of the enmity links. Note that the relative rates do not add up to one because several triads may be closed simultaneously in a single link creation event.

The friendship triadic closure process (FTC), labelled *I* in Fig. 2, is known to play a key role in social network formation [16]. It can be experessed as “The friend of my friend is my friend”, meaning that new friendships tend to be made between people who already have a common friend. Note that the mixed triadic closure process (MTC) *VIII*, is equally important. The phrase “The enemy of my friend is my enemy” is a possible verbalization of this process, and reflects the fact that a person *A* will more likely declare another person *B* as an enemy if already one of *A*’s friends (person *C*) considers *B* as an enemy. Further, if we regard enmity as a psychological reaction to hostile or aggressive behaviour [22], this process can be viewed as an elementary representation of “solidary behavior”.

We quantify the absolute occurrences of the processes FTC and MTC by determining the triadic closure parameters [13, 16], i.e., how often a newly created link closes *at least* one triad of a specific type. We obtain the values $s_\mathrm{f}=0.588$ (FTC). This confirms similar measurements in [13, 16]. We find $s_\mathrm{e}=0.406$ (MTC).

In PARDUS, there are two ways to delete a link: either a link is actively withdrawn by the person that generated it, or a player leaves the game and is removed together with their links from the network. We find that there is a major difference in how often friendship and enmity links are actively deleted. As illustrated in Fig. 2c, friendship links usually last until one of the players leaves the game (only a fraction of $r_\mathrm{f}=0.352$ is actively deleted), while enmity links primarily disappear because players actively delete them ($r_\mathrm{e}=0.894$).

## Model

Based on the network formation analysis, we propose a model for coupled friendship and enmity dynamics that includes the key processes triadic closure, active link deletion, and the addition and removal of nodes. It is a generalization of the model introduced in [16], which is centered around the triadic closure process for reciprocal, positive social relations (friendship). By including uni-directional negative relations (enmity) we take into account the coupled nature of social interactions.

We start with a social network of *N* nodes representing individuals and two network layers representing friendship and enmity relations among the avatars. The network is initialized by sequentially assigning each node two friendship links (undirected) and two enmity links (one incoming, one outgoing) to randomly chosen nodes. Then the following steps are iterated. The dynamics from timestep *t* to $t+1$ is given by:

1. With probability *p*, add a friendship link to the network:
  (1.1) *Friendship triadic closure*. With probability $s_\mathrm{f}$, pick a node with sufficiently large friendship neighborhood (degree $k_\mathrm{f}\ge 2$) at random and connect two randomly selected friends.
  (1.2) With probability $1-s_\mathrm{f}$, pick a node (degree $k_\mathrm{f}\ge 1$) at random and connect one of its friends with any randomly chosen node.
2. With probability $1-p$, add an enmity link to the network:
  (2.1) *Mixed triadic closure*. With probability $s_\mathrm{e}$, pick a node with friendship degree $k_\mathrm{f}\ge 1$ and enmity out-degree $k_\mathrm{e}^\mathrm{out}\ge 1$ at random and randomly select one neighbor in each layer (i.e., one friend and one enemy). Connect the friend with the enemy in such a way that the new link points towards the enemy.
  (2.2) With probability $1-s_\mathrm{e}$, pick a node (degree $k_\mathrm{e}^\mathrm{out}\ge 1$) at random and connect one of its enemies with any randomly chosen node in such a way that the new link points towards the enemy.
3. *Active friendship link deletion*. With probability $r_\mathrm{f}^{*}$, pick a node (degree $k_\mathrm{f}\ge 1)$ at random and remove one of its friendship links.
4. *Active enmity link deletion*. With probability $r_\mathrm{e}^{*}$, pick a node (degree $k_\mathrm{e}^\mathrm{out}\ge 1)$ at random and remove one of its outgoing enemity links.
5. *Node turnover*. With probability *q*, pick a node at random and remove it from the network along with all its links. Introduce a new node and link it to two randomly selected nodes (one friendship link and one incoming enmity link). Then continue with timestep $t+1$.

## Results

### Calibration

The model is completely specified by the set of parameters $(N,p,s_\mathrm{f},s_\mathrm{e},r_\mathrm{f}^{*},r_\mathrm{e}^{*},q)$. All these parameters can be measured from the data in the game. The model has no free parameters. For $q>0$ nodes have a finite lifetime, hence the coupled friendship and enmity dynamics approach a stationary state. Note that nodes enter and leave the network with similar rates. To calibrate the model, we resort to our numerical analysis of the stationary properties of the PARDUS networks (see previous section).

The triadic closure parameters can be directly measured and are $s_\mathrm{f}=0.588$ and $s_\mathrm{e}=0.406$. From the stationarity condition $\Delta L^{+}\approx \Delta L^{-}$ we get that the absolute rates of active link deletion $r_\mathrm{f}^{*}$ and $r_\mathrm{e}^{*}$ and the fractions of actively deleted links $r_\mathrm{f}=0.352$ and $r_\mathrm{e}=0.894$, are related by $r_\mathrm{f}^{*}=p r_\mathrm{f}$ and $r_\mathrm{e}^{*}=(1-p) r_\mathrm{e}$, respectively. Further, stationarity implies:

3 $$\begin{aligned} p&=q (L_\mathrm{f}/N-1)+p r_\mathrm{f},\end{aligned}$$

4 $$\begin{aligned} 1-p&=q (2 L_\mathrm{e}/N-1)+(1-p) r_\mathrm{e} \end{aligned}$$

with $L_\mathrm{f}$ and $L_\mathrm{e}$ denoting the number of friendship links and enmity links, respectively. To see this, consider the respective impact of possible events during a single timestep on the total link balance: the expected number of added friendship links is *p* (FTC or random) plus *q* (node turnover), while $p r_\mathrm{f}$ (active link deletion) plus $q L_\mathrm{f}/N$ (node turnover) links are removed on average. In an analogous manner, a balance equation for enmity links can be found (note the factor 2 due to directionality). We set $L_\mathrm{f}=39,095$, $L_\mathrm{e}=25,699$ and $N=4,232$ as measured on the last day of the observation period. *p* and *q* can be calculated from Eqs. (3) and (4). We obtain $p=0.118$ and $q=0.009$.

We perform numerical simulations of the model using $T=10^6$ timesteps, which correspond to approximately 100 days in the game. To exclude transient effects, we check the stationarity conditions Eqs. (1) and (2) within the final 100 iterations. Results are averaged over 50 realizations. The model is implemented in such a way that previously existing links are overwritten when a new link is created. To avoid the creation of loops in the network, which is very unlikely but can happen in steps 1.2, 2.2 and 5 of the algorithm, a small modification is necessary: If a loop would be created, choose another node. Finally, we tested the network analysis and the simulation outcomes for robustness with respect to the particular choice of the observation period. No significant differences from the presented results were observed with other periods.

### Test of social balance

As in [13], we use a method to empirically test social balance in networks of positively ($+$) and negatively (−) connoted social relations, in particular friendship and enmity. To that end, all links of the network are symmetrized and a triad count is performed for each of the four different types ($+++$,$++-$,$+--$, $---$). The obtained numbers are then compared to the expected numbers in a null model (randomly reshuffled link types), and the *z* score is applied for each triad type (see Methods section).

Social balance theory states that $+++$ triads (‘The friend of my friend is my friend’) and $+--$ triads (‘The enemy of my enemy is my friend’) are stable or balanced, whereas triads of type $++-$ (‘The friend of my friend is my enemy’) are unbalanced and associated with social stress [18, 19, 21]. The purely negative triad $---$ is considered to be unbalanced [19] or balanced [23], depending on the formulation of the theory. People tend to change their relations in such a way that the energy needed for maintaining contacts is minimized [24], and unbalanced triads are avoided. As a consequence, balanced triads are expected to be over-represented in the network (positive *z* score), and unbalanced triads are expected to be under-represented (negative *z* score).

We use this method to test both the PARDUS data at the final day of the observation period, and the averaged simulation outcomes. We find that $+++$ and $+--$ triads are heavily over-represented both in the data and in the model. For $+++$ we find $z$ scores of $10$ for the model and $71$ for the data. For $+--$ we get $-105$ for the model and $-112$ for the data, for $+--$ we have $113$ for the model and $47$ for the data, and finally for $---$ we have $1$ for the model and $-5$ for the data.

We conclude that the friendship and enmity networks in PARDUS are compatible with social balance theory in its weak formulation [23]. In [13], similar results were reported. We further see that the model endogenously leads to social balance.

### Network properties: data and model

**Fig. 3:**
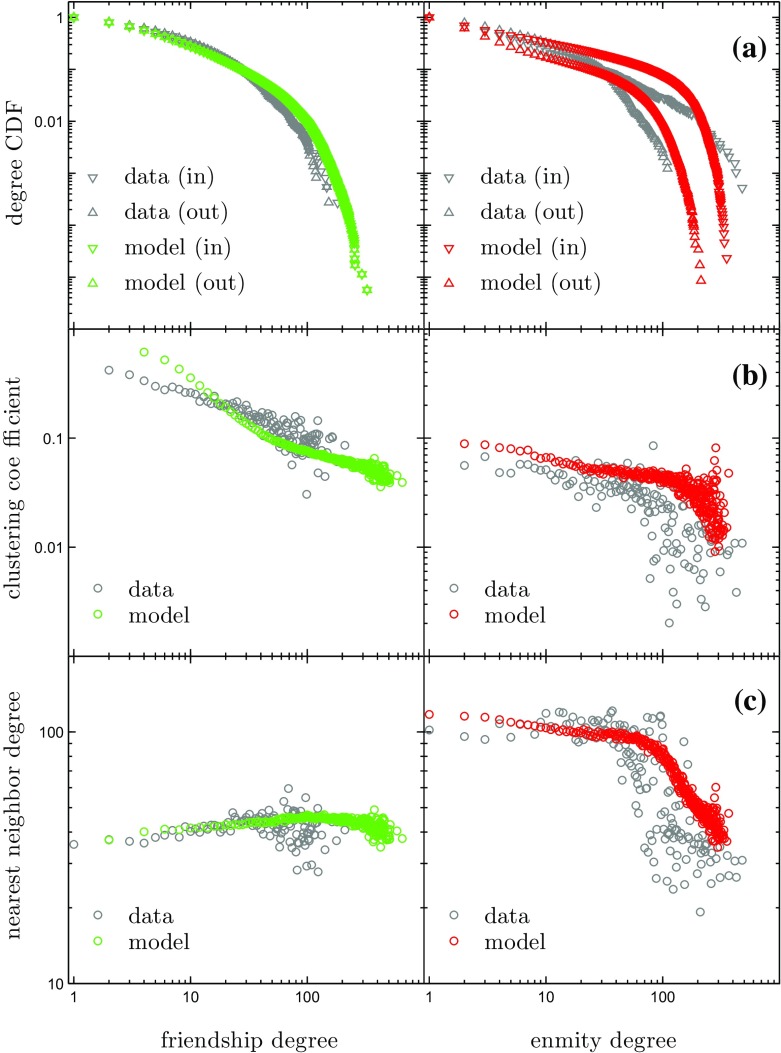
Properties of the coupled two-layer friendship (left) and enmity (right) networks obtained from the PARDUS data (grey) and model simulations (green and red): **a** cumulative distributions of in-degrees ($\nabla$) and out-degrees ($\Delta$), **b** clustering coefficient as a function of degree, **c** nearest neighbor degree versus degree. To evaluate **b** and **c**, the networks were symmetrized

We now validate the model by calculating the distributions of degrees, clustering coefficients and nearest neighbor degrees for the simulated networks. We compare them to the actual distributions in the PARDUS networks at the final day of the observation period.

Figure 3a shows the cumulative in-degree and out-degree distributions for the friendship (left) and enmity (right) networks. We observe that the friendship degree distributions from the data (grey) and the model (green) are in good agreement. Note that the model only allows for bidirectional friendship links. Therefore the in-degree ($\nabla$) and out-degree ($\Delta$) distributions are identical. The enmity in-degree distribution of the data shows an approximate power law behavior with an exponent close to $-1$. Such a behavior is absent in the enmity out-degree distribution of the data. The model distributions initially follow a power law with exponents close to $-1$, before rapidly decreasing for large degree values. While the model overestimates the true distribution values in the middle range of degrees (approximately between 30 and 110), it does account for the asymmetry in enmity in- and out-degrees and the power law behavior.

Figure 3b depicts the clustering coefficient of the symmetrized friendship (left) and enmity (right) networks as a function of degree. In both cases, we find good agreement between data and model. In the friendship networks, the clustering coefficients decrease with increasing degree, following a power law. The enmity clustering coefficient versus degree exhibits a weak downward trend (approximate power law) for degrees up to approximately 100, and then starts to fluctuate with average values dropping rapidly. This drop is likely to be related to the asymmetry of the cumulative enmity degree distributions, in particular to the existence of nodes with a large in-degree and a comparatively small out-degree, public enemies [14, 15].

The nearest neighbor degree versus degree functions can be seen in Fig. 3c. There is a considerable overlap between data and model. The friendship nearest neighbor degrees are to a large extent independent of the degrees (uniform distribution). The distributions of the enmity nearest neighbor degrees are also close to uniform for most degrees, but follow an approximate power law downward trend for degrees larger than about 100. This is captured well in the model.

## Discussion

The conjecture that structural and dynamical properties of social networks are intimately connected, and that this connection is encoded largely in triadic processes involving positively and negatively connoted relations, dates back as far as Heider’s balance theory [19]. It is surprising that the two essential questions in this context have been addressed so poorly up to now, namely: What are the key processes behind social network formation? And, how do these processes lead to structural network properties? This can be partially explained by limited data availability especially for negatively connoted relations. The main finding in this paper is that it is possible to understand the dynamics of positively connoted relations on a standalone basis [16]. However, this does not apply for negatively connoted relations.

Based on a numerical analysis of social network data for friendship and enmity relations from the massive multiplayer online game PARDUS, we can identify five key processes of social network formation: friendship triadic closure (FTC), mixed triadic closure (MTC), active friendship link deletion, active enmity link deletion, and node turnover. FTC and node turnover constitute an autonomous description of friendship link dynamics. This explains the success of the model in [16], which provides an understanding of positive-connoted links only. In contrast, due to the mixed nature of the MTC process the dynamics of enmity links strongly depends on the friendship network.

To clarify how microscopic processes lead to the structural network properties, we proposed a simple model for network formation based on the identified five key processes. The triadic closure processes FTC and MTC naturally feature a preferential attachment mechanism, since the probability to choose the neighbor of a randomly selected node is an increasing function of its in-degree [25]. However, the joint friend and enmity link dynamics leads to rich network structures, including non-trivial behavior in the degree distributions. Also the clustering and nearest neighbor degrees, when plotted against the degree, show behavior that was so-far not understood. The model does neither control the multiplicity nor the co-occurrence rates of triadic closure events, only the rate at which at least one triad of one type is closed.

We find surprisingly good agreement between the model results and the original PARDUS data in terms of these structural network properties. The same is true for the *z* scores of the four basic types of mixed triads. The results can be interpreted both as a validation of the model and as evidence for its explanatory power.

In [13] an alternative model for social network formation based on triadic closure was offered. However, in contrast to the model presented here, it lacks essential features including the directionality of enmity links which are necessary for asymmetrical degree distributions and specific link deletion mechanisms.

## Methods

We calculate the overlap between friendship and enmity networks by means of the *Jaccard coefficient*,

5 $$\begin{aligned} J(F,E) = \frac{\left| F\cap E\right| }{\left| F \cup E\right| }, \end{aligned}$$

where *F* and *E* are the respective sets of links. Further, we measure *reciprocity* by,

6 $$\begin{aligned} \rho = \frac{r^{*}-\bar{a}}{1-\bar{a}}, \end{aligned}$$

where $r^{*}$ is the fraction of links that are in mutual dyads and $\bar{a}$ is the ratio of observed to possible links [26].

To test for social balance, we follow the method introduced in [13]. First, we symmetrize the friendship ($+$) and enmity (−) links and remove links that are in both networks. We perform a triad count for each of the four types ($+++$,$++-$,$+--$,$---$). Next, we construct a null model for the respective numbers of triads. To that end, we reshuffle the link types without changing the topology, i.e., we randomly assign link types $+$ and − to the existing links, while keeping the number of $+$ and − links fixed. We then count the triads of each type again. We repeat this step until 1000 realizations of the null model are obtained. Finally, we calculate the *z* score,

7 $$\begin{aligned} z = \frac{N-\bar{N}}{\sigma } , \end{aligned}$$

for each of the triad types, where *N* is the observed number of triads of a certain type, $\bar{N}$ the average number in the null model and $\sigma$ the standard deviation of the null model.
